# MAPK Phosphatase-1 Deficiency Exacerbates the Severity of Imiquimod-Induced Psoriasiform Skin Disease

**DOI:** 10.3389/fimmu.2018.00569

**Published:** 2018-03-21

**Authors:** Weiheng Zhao, Shuxiu Xiao, Hongjin Li, Tingting Zheng, Jian Huang, Ran Hu, Baohua Zhang, Xinguang Liu, Gonghua Huang

**Affiliations:** ^1^Hongqiao International Institute of Medicine, Shanghai Tongren Hospital, Faculty of Basic Medicine, Shanghai Institute of Immunology, Key Laboratory of Cell Differentiation and Apoptosis of Chinese Ministry of Education, Shanghai Jiao Tong University School of Medicine, Shanghai, China; ^2^Guangdong Provincial Key Laboratory of Medical Molecular Diagnostics, Guangdong Medical University, Dongguan, China

**Keywords:** mitogen-activated protein kinase, phosphatase MAPK phosphatase-1, psoriasis, macrophage, keratinocyte

## Abstract

Persistent activation of mitogen-activated protein kinase (MAPK) is believed to be involved in psoriasis pathogenesis. MAPK phosphatase-1 (MKP-1) is an important negative regulator of MAPK activity, but the cellular and molecular mechanisms of MKP-1 in psoriasis development are largely unknown. In this study, we found that the expression of MKP-1 was decreased in the imiquimod (IMQ)-induced psoriasiform mouse skin. MKP-1-deficient (MKP-1^−/−^) mice were highly susceptible to IMQ-induced skin inflammation, which was associated with increased production of inflammatory cytokines and chemokines. MKP-1 acted on both hematopoietic and non-hematopoietic cells to regulate psoriasis pathogenesis. MKP-1 deficiency in macrophages led to enhanced p38 activation and higher expression of interleukin (IL)-1β, CXCL2, and S100a8 upon R848 stimulation. Moreover, MKP-1 deficiency in the non-hematopoietic compartments led to an enhanced IL-22 receptor signaling and higher expression of CXCL1 and CXCL2 upon IMQ treatment. Collectively, our data suggest a critical role for MKP-1 in the regulation of skin inflammation.

## Introduction

Inflammation is important for host to protect against infection and injury (1). However, uncontrolled inflammation seems to be pathogenic relevance to many inflammatory diseases (2). Psoriasis is a chronic inflammatory skin disease that is affecting around 2% of the world’s population (3–5). The major clinical manifestations of psoriasis are characterized by epidermal hyperplasia, erythematous plaque formation, and the inflammatory cell infiltration in the dermis and epidermis (3, 4). Notably, patients with psoriasis are highly associated with many systemic inflammatory diseases, such as diabetes and cardiovascular disease (4). Although the precise molecular mechanism of psoriasis is incompletely understood, accumulating evidence indicates that the dysfunction of the immune system plays crucial roles in its development (4). Recently, substantial studies have identified a central role of pro-inflammatory cytokines, including tumor necrosis factor α (TNF-α), interleukin (IL)-1β, IL-6, IL-23, and IL-17, in psoriasis pathogenesis (6). Indeed, the use of cytokine antagonists is an important therapeutic strategy in the current treatment of psoriasis (7), although the molecular signaling pathways that regulate those key cytokines are not well described. Thus, further dissection of the signaling pathways that regulate the altered psoriatic gene expression is essential for uncovering the psoriasis pathogenesis and better treatment of this disease.

The mitogen-activated protein kinase (MAPK) pathway is one of the most important central pathways that respond to extracellular signals and transduce the signals to further direct the expression of various cytokines and chemokines in the cells (8). The MAPK family has four subfamilies, including the extracellular signal-regulated kinases (ERK1/2), c-Jun NH2-terminal kinases (JNK-1/2/3), p38 (p38α/β/γ/δ), and ERK5 (8). Recent studies have shown that compared with non-lesional psoriatic skin, the activities of ERK and p38 MAPK are increased in lesional psoriatic skin, while JNK activity has been found unaltered in most studies (9, 10). The p38 MAPK-dependent antimicrobial peptide (AMP) S100A8 is also found upregulated in lesional psoriatic skin (11). Moreover, the clinical efficacy by using adalimumab (anti-TNF-α) therapy in psoriatic patients is mainly through inhibiting p38 activity to further reduce expression of certain p38 kinase-regulated pro-inflammatory cytokines, indicating that targeting p38 could be a promising strategy to treat psoriasis (12). Now it is widely believed that persistent activation of MAPK signaling pathway is pathogenic relevance to psoriasis and a tight regulation of MAPK activity is essential for maintaining a beneficial level of skin homeostasis during the inflammation or injury (13).

Negative regulation of MAPK activity is achieved mainly by MAPK phosphatases (14). MAPK phosphatase-1 (MKP-1) (also named as dual-specificity phosphatase 1) is mainly localized to the nucleus and preferentially dephosphorylates p38 and JNK (15). Previous studies have shown that MKP-1 negatively regulates TLR-induced innate immune responses and MKP-1^−/−^ mice show highly susceptibility to endotoxic shock, which is associated with increased production of pro-inflammatory cytokines and mediators (8). LPS-stimulated MKP-1^−/−^ macrophages have increased and sustained activation of p38 and JNK, and produce large amounts of pro-inflammatory cytokines, including TNF-α, IL-6, and IL-10, as well as chemokines such as CCL3, CCL4, and CXCL2 (16). Recent studies have shown that the roles of MKP-1 in immunoregulation are cell type dependent. MKP-1 could program dendritic cell (DC)-derived T cell polarizing cytokines to instruct Th1, Th17, and iTreg cell differentiation and T cell-mediated adaptive immune responses, thus bridging innate and adaptive immunity to coordinate protective immunity and immunopathology (17). Moreover, MKP-1 has been shown to play an important role in promoting T cell activation, proliferation, and function (18). Notably, in psoriatic skin lesions, MKP-1 mRNA expression is significantly downregulated compared with the non-lesional psoriatic skin (19). Considering that MAPK activity is increased in psoriasis lesions and MKP-1 is the major negative regulator of MAPK pathway, the decreased MKP-1 expression might indicate an essential role of MKP-1 in the pathogenesis of psoriasis, but the underlying cellular and molecular mechanisms are still unknown.

To explore the role of MKP-1 in psoriasis pathogenesis, we employed the recently described imiquimod (IMQ)-induced psoriasiform skin disease model (20). IMQ is a TLR7/8 agonist that has been widely used to treat certain virus-associated and malignant skin diseases (21). IMQ can induce *de novo* and exacerbate preexisting psoriasis lesions in human (22), and daily application of IMQ cream can induce mouse dermatitis with many features resembling human psoriasis (20). In the current study, we found that MKP-1^−/−^ mice were highly susceptible to IMQ-induced psoriasiform skin inflammation. The disease severity was partially associated with enhanced p38 activity and increased IL-1β, CXCL2, and S100A8 expressions in MKP-1^−/−^ macrophages. Moreover, we also found an increased IL-22 receptor signaling and higher expression of CXCL1 and CXCL2 in MKP-1^−/−^ non-hematopoietic compartments [mainly composed of keratinocytes (KCs)] upon IMQ treatment. These findings demonstrate a potential clinical usefulness by modulating MKP-1 activity in psoriasis.

## Materials and Methods

### Mice and Bone Marrow (BM) Chimeras

MKP-1^−/−^ mice have been described previously (23). C57BL/6 mice were from Shanghai SLAC Laboratory Animal Center. All mice had been backcrossed to C57BL/6 background for at least 8 generations. Age- and sex-matched mice at 6–10 weeks of age were used for all experiments. For *in vivo* IMQ-induced disease assay, there were at least three mice per group. For non-hematopoietic chimeric experiments, BM cells from wild-type (WT) mice (CD45.1^+^) were intravenously transferred into lethally irradiated WT or MKP-1^−/−^ mice (CD45.2^+^) (7.5 × 10^6^ BM cells/recipient). For hematopoietic chimeric experiments, BM cells from WT or MKP-1^−/−^ mice (CD45.2^+^) were intravenously transferred into lethally irradiated WT mice (CD45.1^+^) (7.5 × 10^6^ BM cells/recipient). All mice were kept in specific pathogen-free conditions in the Animal Resource Center at Shanghai Jiao Tong University School of Medicine. This study was carried out in accordance with the recommendations of the Care and Use of Laboratory Animals with the approval (SYXK-2003-0050) of the Scientific Investigation Board of Shanghai Jiao Tong University School of Medicine, the Institutional Animal Care and Use Committee of Shanghai Jiao Tong University School of Medicine. The protocol was approved by the Institutional Animal Care and Use Committee of Shanghai Jiao Tong University School of Medicine.

### IMQ-Induced Mouse Psoriasiform Skin Disease Model

A dose of 25 mg cream containing 5% IMQ (MedShine) was topically applied to per ear of each mouse daily, and the dose of Vaseline (Fagron) was applied to the control group for five consecutive days. Ear thickness was measured using a micrometer and averaged each day according to previously described (24) by two experienced experimenters. Ear pictures were taken.

### Pharmacological Inhibition of p38

Imiquimod-treated WT and MKP-1^−/−^ mice were intraperitoneally administrated with p38 inhibitor SB203580 (Merck CalBiochem) at a dose of 0.75 mg/kg body weight for five consecutive days. For *in vitro* macrophage treatment, cells were incubated with vehicle or 10 μM SB203580 (Merck Calbiochem) for 0.5 h before stimulation.

### Cell Culture and Purification

Total BM cells were flushed with PBS from mouse femurs and tibiae. For BM-derived DC (BMDC) culture, BM precursors were cultured in RPMI-1640 medium supplemented with 10% FBS (vol/vol), 10 ng/ml recombinant murine granulocyte-macrophage colony-stimulating factor (GM-CSF, R&D), and 4 ng/ml recombinant murine IL-4 (R&D). Non-adherent cells were removed and fresh BMDC culture medium with GM-CSF and IL-4 was added at day 3. Mature DCs were harvested for analysis at day 7. For BM-derived macrophage (BMDM) culture, BM cells were cultured in DMEM medium supplemented with 10% FBS (vol/vol) and 15 ng/ml recombinant murine macrophage colony-stimulating factor (M-CSF, R&D). Half of the medium was changed with prewarmed fresh medium with M-SCF after 60 h. Mature macrophages were harvested for analysis at day 5. For neutrophil isolation, BM cells were suspended in 45% percoll (GE Healthcare) and laid on the top of 62% and 81% percoll gradient, and then centrifuged at 1,500 × *g* for 30 min at room temperature. Mature neutrophils were collected from the interface of 62% and 81% percoll.

### Skin Cell Preparation

Mouse skin samples were dissected from the ear of mice. After a thorough rinse, ears were split into dorsal and ventral halves, then the subcutaneous fat tissue was carefully scraped off and ears were floated split side down for 40 min at 37°C on the surface of 0.5% Trypsin (vol/vol) (Gibco). The dermis was separated from the epidermis. Each sheet was cut into small pieces and placed into digestion solution containing 1.5 mg/ml (for dermis) or 1 mg/ml (for epidermis) collagenase IV (Gibco). Digestion was performed for 90 min (for dermis) or 80 min (for epidermis) at 37°C with brief mixing. After the digestion, the solution was mixed thoroughly and filtered through a nylon filter to obtain single-cell suspension.

### Flow Cytometry

For analysis of surface markers, cells were stained in PBS/1% FBS with anti-CD45 (30-F11), anti-CD11b (M1/70), anti-Gr-1 (RB6-8C5), anti-F4/80 (BM8), anti-CD11c (N418), anti-MHC-II (M5/114.15.2), anti-γδTCR (eBioGL3), anti-CD3 (17A2), anti-CD4 (RM4-5), anti-CD8α (53-6.7), and anti-TCRβ (H57-597) (all from eBioscience). For intracellular staining with anti-IL-17 (eBio17B7) and anti-interferon (IFN)-γ (XMG 1.2) (all from eBioscience), cells were stimulated with PMA and ionomycin in the presence of protein transport inhibitor for 5 h before being stained according to the manufacturer’s instructions (BD Biosciences). Anti-Foxp3 (FJK-165, eBioscience) staining was done according to the manufacturer’s instructions (eBioscience). Flow cytometry data were acquired on BD FACSCannto™ II or BD LSRFortessa™ X-20 and analyzed with FlowJo software (Treestar). KCs were sorted with a BD FACSAria™ III Sorter.

### Histopathological Analysis

Formalin-preserved mouse ear sections were embedded in paraffin according to standard techniques. Longitudinal sections were stained with hematoxylin and eosin and analyzed by microscopic examination.

### RNA and Protein Analyses

Total RNA of skin tissue and cells was isolated using the Trizol reagent (Invitrogen) and RNeasy mini Kit (GeneMark), respectively. Reverse transcription was performed *via* PrimeScript RT MasterMix (TAKARA) according to the manufacturer’s instructions. Quantitative PCR was carried out with SYBR Green PCR Master Mix (Applied Biosystems) in a Vii7 Real-Time PCR system (Applied Biosystems). Relative mRNA levels were determined with HPRT as a reference gene. The primer sequences were used as indicated in Table 1. For cytokine detection in skin tissue, 45 mg skin tissue were weighted and homogenized in 0.5 ml ice-cold CelLytic™ MT Cell Lysis Reagent (Sigma-Aldrich). Concentration of CXCL2, IL-1β, and IFN-γ in homogenized supernatants was measured by ELISA according to the manufacturer’s instructions (eBioscience). Immunoblot analyses were performed as described (25) with antibody to p38 phosphorylated at Thr180 and Tyr182 (D3F9), antibody to ERK phosphorylated at Thr202 and Tyr204 (D13.14.4E), and antibody to IκBα phosphorylated at Ser32 (14D4) (all from Cell Signaling Technology) and GAPDH (Proteintech).

**Table 1:** Primers used in quantitative RT-PCR.

Primers	Sequence (5′–3′)	
HPRT	Fw	TCAGTCAACGGGGGACATAAA
	Rv	GGGGCTGTACTGCTTAACCAG
Interleukin (IL)-17	Fw	TGCTACTGTTGATGTTGGGAC
	Rv	AATGCCCTGGTTTTGGTTGAA
Tumor necrosis factor α	Fw	CAGGCGGTGCCTATGTCTC
	Rv	CGATCACCCCGAAGTTCAGTAG
IL-1β	Fw	GCAACTGTTCCTGAACTCAACT
	Rv	ATCTTTTGGGGTCCGTCAACT
CSF2	Fw	GGCCTTGGAAGCATGTAGAGG
	Rv	GGAGAACTCGTTAGAGACGACTT
CXCL1	Fw	TAGGGTGAGGACATGTGTGG
	Rv	AAATGTCCAAGGGAAGCGT
CXCL2	Fw	CCAACCACCAGGCTACAGG
	Rv	GCGTCACACTCAAGCTCTG
Interferon-γ	Fw	GCCACGGCACAGTCATTGA
	Rv	TGCTGATGGCCTGATTGTCTT
IL-10	Fw	CTTACTGACTGGCATGAGGATCA
	Rv	GCAGCTCTAGGAGCATGTGG
IL-22	Fw	ATGAGTTTTTCCCTTATGGGGAC
	Rv	GCTGGAAGTTGGACACCTCAA
IL-22r	Fw	GCTGGACTCCCTTGTGTGT
	Rv	CACATGGCCTCAGTCTCAA
S100A7	Fw	GCCTCCTACATGGACAAAGTC
	Rv	GCTTCTCGTACCACTCCTTGA
S100A8	Fw	TGTCCTCAGTTTGTGCAGAATATAAA
	Rv	TCACCATCGCAAGGAACTCC
Keratin 10	Fw	GGAAGCCTCCTTGGCAGAA
	Rv	GCGGAGATCTGGCTTTGAATC
Loricrin	Fw	CTCCTGTGGGTTGTGGAAAGA
	Rv	TGGAACCACCTCCATAGGAAC
Dual-specificity phosphatase 1	Fw	CTCCAAGGAGGATATGAAGCG
	Rv	CTCCAGCATCCTTGATGGAGTC

### Statistical Analysis

The data were analyzed with GraphPad Prism 5 and are presented as the mean ± SEM. Student’s *t*-test was used when two conditions were compared. Probability values were indicated and of <0.05 were considered significant; two-sided Student’s *t*-tests were performed (ns, not significant).

## Results

### MKP-1^−/−^ Mice Are Hypersusceptible to IMQ-Induced Psoriasiform Skin Disease

**Figure 1:**
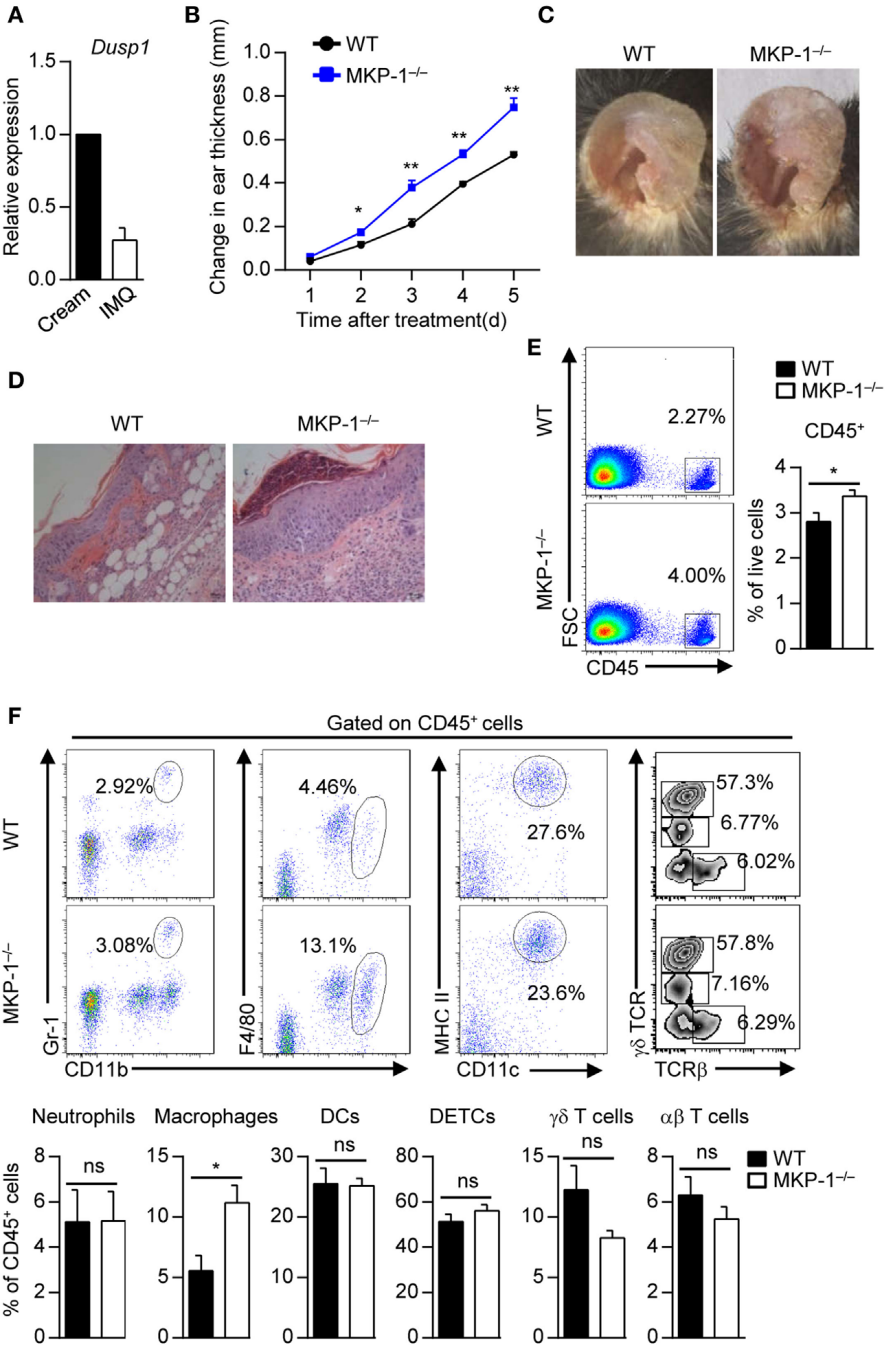
MKP-1^−/−^ mice are hypersusceptible to imiquimod (IMQ)-induced psoriasiform skin disease. **(A)** Wild-type (WT) mice were topically treated with IMQ-containing cream or control cream for five consecutive days, and the expression of MAPK phosphatase-1 (MKP-1) mRNA (*Dusp1*) in the skin was analyzed by real-time PCR. *n* = 3 mice per group. **(B–E)** WT and MKP-1^−/−^ mice were topically treated with IMQ for five consecutive days. *n* = 4–6 mice per group. Ear thickness was daily measured using a micrometer to calculate ear swelling **(B)**, and picture was taken on day 5 **(C)**. Histopathological changes in skin tissue were detected by hematoxylin and eosin staining (scale bars: 50 µm) **(D)**. Infiltration of indicated cell populations in the epidermis was analyzed by flow cytometry **(E,F)**. Dendritic epidermal T cell (DETC). Data are presented as mean ± SEM. Data are representative of two to three independent experiments.

Although MKP-1 mRNA expression in psoriatic skin lesions is lower than that in non-lesional psoriatic skin (19), the involved cellular and molecular mechanisms of MKP-1 in psoriasis pathogenesis are still largely unknown. In the IMQ-induced mouse psoriasiform skin disease model, there was much lower MKP-1 expression in IMQ-treated mouse skin than control cream-treated mouse skin (Figure 1A). Further studies showed that this downregulation of MKP-1 mRNA levels started from day 2, a time point preceding the development of dermatitis, and was correlated with an increased expression of p38α in the IMQ-treated skin (Figure S1A in Supplementary Material). Interestingly, we did not observe this downregulation of MKP-1 mRNA expression in the draining lymph nodes (DLNs) upon IMQ treatment (Figure S1B in Supplementary Material), as well as in skin tissue of 2,4-dinitrofluorobenzene-mediated Th1-type or fluorescein isothiocyanate-mediated Th2-type dermatitis (Figures S1C,D in Supplementary Material), indicating that the regulation of MKP-1 mRNA levels shows certain specificity in different stimuli stimulation or different disease models.

To study the role of MKP-1 in psoriasis pathogenesis, we topically treated WT and MKP-1^−/−^ mice with IMQ-containing cream. We found that MKP-1^−/−^ mice were highly susceptible to the pathological characteristics of psoriasis, including ear swelling, epidermal hyperplasia and skin inflammatory cell infiltration (Figures 1B–D). To further explore the cellular mechanism affected by MKP-1, we characterized and quantitated the inflammatory cell infiltration in the epidermis by flow cytometry. MKP-1^−/−^ mice had significantly higher infiltration of CD45^+^ leukocytes and macrophages in the epidermis than WT mice, while the neutrophils, conventional DCs and T cells were comparable between WT and MKP-1^−/−^ mice (Figures 1E,F). Notably, among the CD11b^+^F4/80^+^ population, parts of cells expressed monocyte surface marker Ly6C (Supplementary Figure S2 in Supplementary Material), indicating that inflammatory monocyte-derived DCs might also play a role during psoriasis development. These results indicate that MKP-1 deficiency correlates with more severe skin inflammation in IMQ-induced psoriasiform model.

### MKP-1 Deficiency Leads to Enhanced Cytokine Production in Skin Tissue

**Figure 2:**
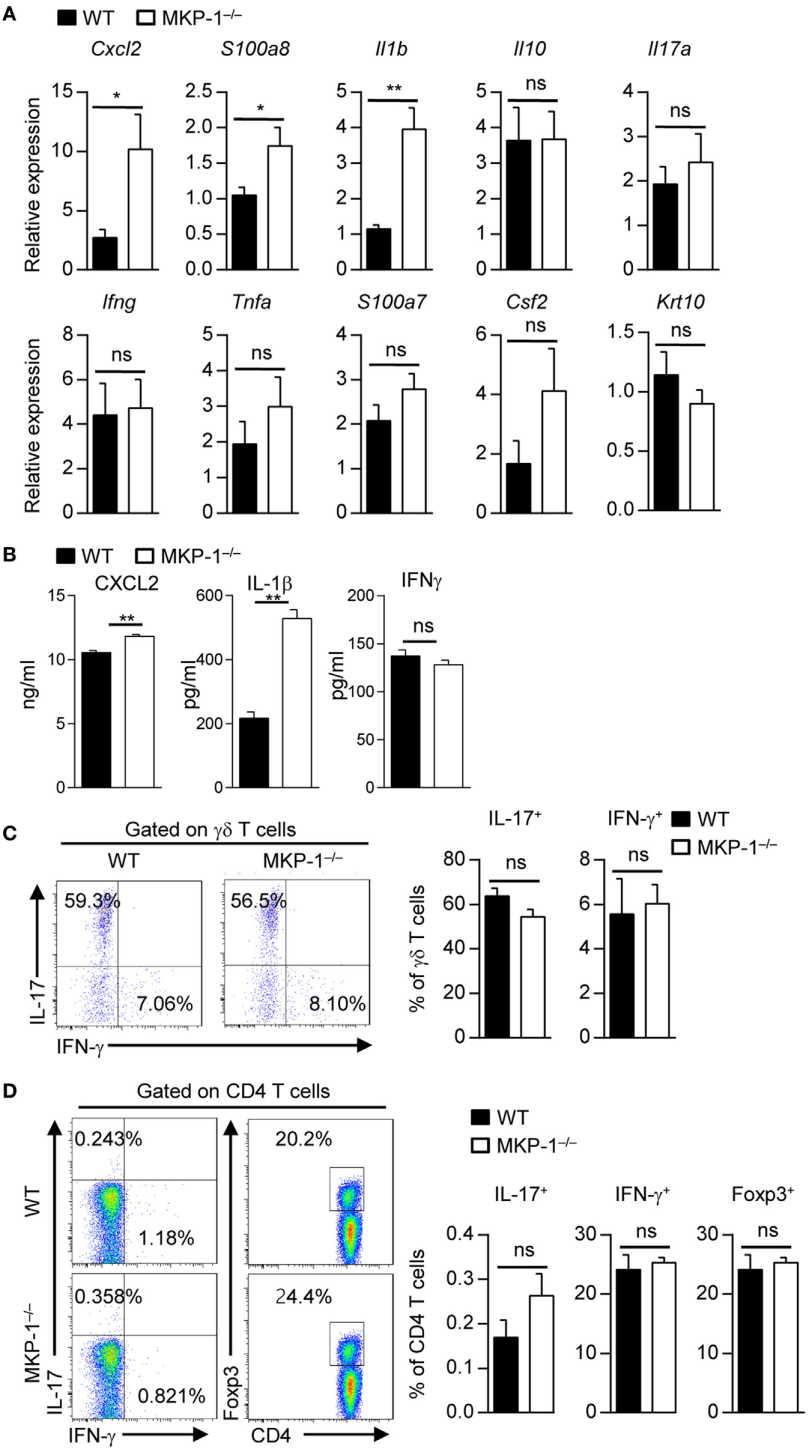
MAPK phosphatase-1 (MKP-1) deficiency leads to enhanced cytokine production in skin tissue. Wild-type (WT) and MKP-1^−/−^ mice were topically treated with imiquimod for five consecutive days. *n* = 4–5 mice per group. **(A)** Relative mRNA expression of inflammatory-related genes in skin tissue. **(B)** Protein levels of CXCL2, interferon (IFN)-γ, and interleukin (IL)-1β in supernatant isolated from mouse ear skin tissue were analyzed by ELISA. **(C)** The percentages of IL-17^+^ γδ T cells and IFN-γ^+^ γδ T cells in the draining lymph nodes (DLNs). **(D)** The percentages of IL-17^+^CD4^+^ T cells, IFN-γ^+^CD4^+^ T cells, and Foxp3^+^CD4^+^ T cells in the DLNs. Data are presented as mean ± SEM. Data are representative of two independent experiments.

We next determined whether MKP-1 could regulate the expressions of inflammatory-related cytokines and chemokines during psoriasis development. Quantitative RT-PCR analysis of inflamed skin from MKP-1^−/−^ mice revealed significantly increased expression of chemokines involved in macrophage attraction (*Cxcl2*) and AMPs typically present in psoriasis lesions (*S100a8*) (Figure 2A). Moreover, mRNA expression of *Il1b*, which is crucially involved in psoriatic skin inflammation (20), was significantly increased in the skin of MKP-1^−/−^ mice (Figure 2A). We also used ELISA to measure certain cytokine production from the supernatant isolated from IMQ-treated mouse skin tissues. The results showed that the protein levels of CXCL2 and IL-1β were significantly higher in MKP-1^−/−^ mice than those in WT mice (Figure 2B), which were consistent with the mRNA levels of those cytokines. IL-17-related cytokines have been shown to play essential roles in psoriasis pathogenesis (6); however, MKP-1^−/−^ mice had comparable expression of *Il17, Ifng*, and *Tnfa* in the skin compared with WT mice. Although there was substantial infiltration of T cells, the majority of which were γδ T cells as previously reported (26), and the percentages of IL-17- and interferon (IFN)-γ-producing γδ and CD4^+^ T cells did not differ between WT and MKP-1^−/−^ mice (Figures 2C,D). Thus, these results indicate that MKP-1 suppresses cytokine production in skin tissues during IMQ-induced psoriasiform skin inflammation.

### MKP-1 Deficiency in Hematopoietic-Derived Cells Exacerbates the Development of IMQ-Induced Psoriasiform Inflammation

**Figure 3:**
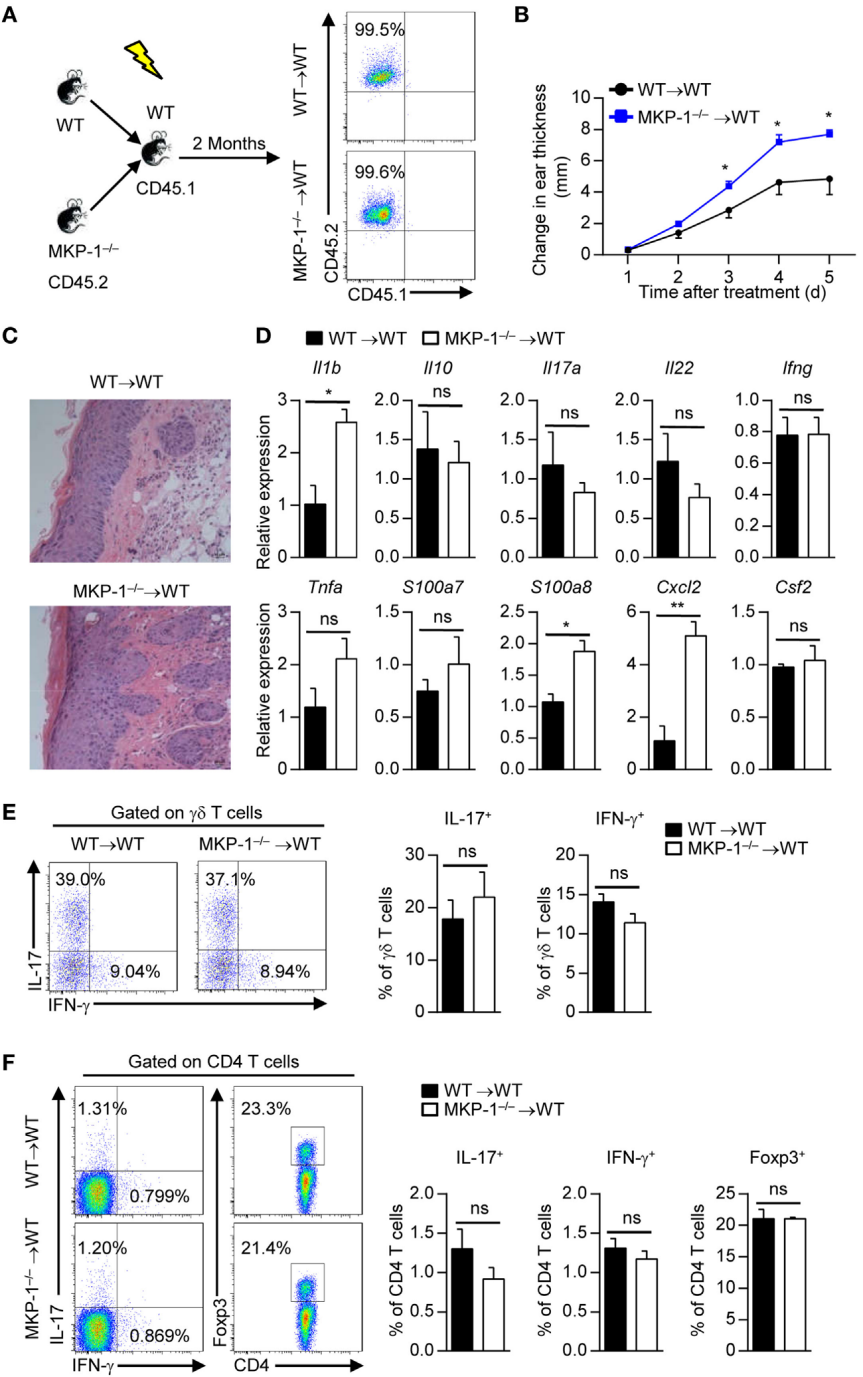
MAPK phosphatase-1 (MKP-1) deficiency in hematopoietic-derived cells exacerbates the development of imiquimod (IMQ)-induced psoriasiform inflammation. Bone marrow (BM) cells of wild-type (WT) and MKP-1^−/−^ mice were transplanted into X-ray-irradiated WT mice to generate the WT → WT and MKP-1^−/−^ → WT chimeras. **(A)** Two months after the generation of BM chimeras, blood cells were analyzed by flow cytometry. **(B–F)** The chimeras were treated with IMQ for five consecutive days. *n* = 4–6 mice per group. Changes in ear thickness **(B)**, representative images of hematoxylin and eosin staining of skin section (scale bars: 50 µm) **(C)**, the relative expression of inflammatory-related genes in skin tissue **(D)**, the percentages of IL-17^+^ γδ T cells and IFN-γ^+^ γδ T cells in the draining lymph nodes (DLNs) **(E)**, and the percentages of IL-17^+^CD4^+^ T cells, IFN-γ^+^CD4^+^ T cells, and Foxp3^+^CD4^+^ T cells in the DLNs **(F)** were analyzed. Data are presented as mean ± SEM. Data are representative of two independent experiments.

To identify whether MKP-1 in hematopoietic-derived cells is responsible for the hyperinflammatory skin response in MKP-1^−/−^ mice, we transplanted BM cells of WT and MKP-1^−/−^ mice into X-ray-irradiated WT mice to generate WT → WT and MKP-1^−/−^ → WT chimeras (Figure 3A). Two months after transplantation, chimeras were treated with IMQ to induce psoriasiform skin disease. Compared with IMQ-treated WT → WT chimeras, MKP-1^−/−^ → WT chimeras showed enhanced ear thickness (Figure 3B). Histological analysis showed that the skin of MKP-1^−/−^ → WT chimeras had significantly higher epidermal hyperplasia and inflammation (Figure 3C). Moreover, skin samples from MKP-1^−/−^ → WT chimeras exhibited higher levels of *Il1b, S100a8*, and *Cxcl2* mRNA expression, but normal expression of *Il17, Tnfa*, and *Infg* mRNA at the peak of disease than those from WT → WT chimeras (Figure 3D). Again, intracellular staining showed comparable IL-17 and IFN-γ production from γδ^+^ and CD4^+^ T cells in the DLNs of both MKP-1^−/−^ → WT and WT → WT chimeras (Figures 3E,F). These results indicate that MKP-1 in hematopoietic compartments regulates the disease severity of IMQ-induced psoriasiform skin inflammation.

### MKP-1 Negatively Regulates Cytokine Expression Through Inhibition of p38 Activity in Macrophages

**Figure 4:**
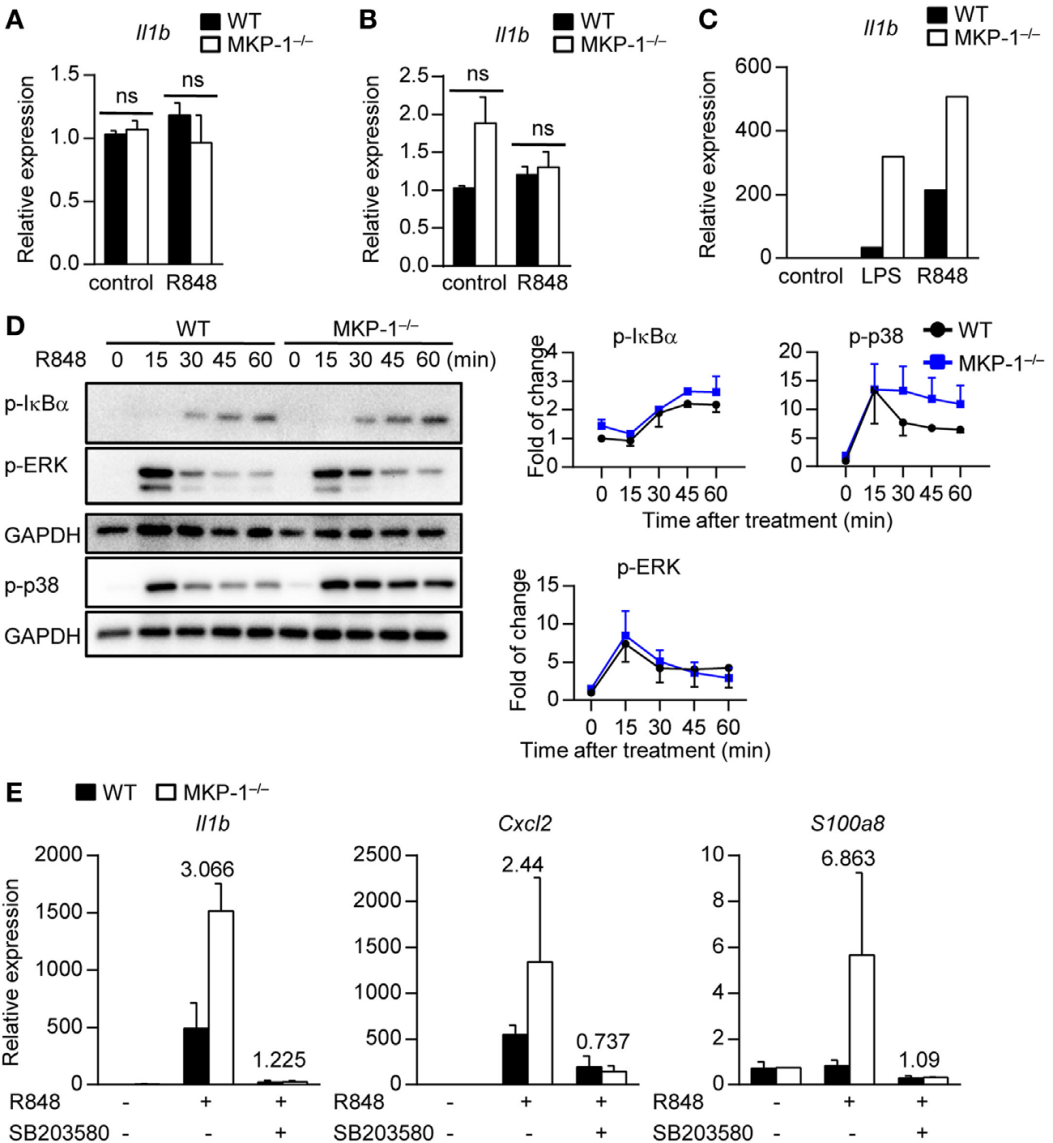
MAPK phosphatase-1 (MKP-1) negatively regulates cytokine expression through inhibition of p38 activity in macrophages. **(A–C)** Bone marrow (BM) cells from wild-type (WT) and MKP-1^−/−^ mice were used for *in vitro* dendritic cell culture, neutrophil isolation, and macrophage culture. *Il1b* mRNA expression was examined in BM-derived DCs **(A)**, neutrophils **(B)**, and BM-derived macrophages **(C)** after being treated with R848 or LPS for 5 h. **(D)** The abundances of phosphorylated IκBα, ERK, and p38 were analyzed by Western blot (left), and the relative expression was normalized by GAPDH (right). **(E)** WT and MKP-1^−/−^ macrophages were treated with R848 for 5 h in the presence or absence of SB203580 to examine the expression of *Il1b, Cxcl2*, and *S100a8*. Data are representative of two independent experiments.

Next, we examined the involved cell types of MKP-1 in hematopoietic compartments *in vitro*. Given DCs have been shown to play an essential role in psoriasis development (27), we stimulated WT and MKP-1^−/−^ BMDCs with TLR7/8 ligands R848 and measured the cytokine expression. We found that both WT and MKP-1^−/−^ DCs had comparable *Il1b* expression upon stimulation (Figure 4A). Neutrophils are the dominant skin infiltrating immune cells during psoriasis development (28); however, MKP-1 activity in neutrophils was not required for the *Il1b* expression upon stimulation (Figure 4B).

An increased macrophage infiltration in IMQ-treated MKP-1^−/−^ mice prompted us to gather further evidence for dysregulated cytokine expressions in MKP-1^−/−^ macrophages. BMDMs from WT and MKP-1^−/−^ mice were stimulated with R848 and cytokine expression was analyzed by real-time PCR. The results showed that the mRNA expression of *Il1b* was higher in MKP-1^−/−^ BMDMs than that in WT BMDMs (Figure 4C), indicating that MKP-1 activity in macrophages might be involved in the pathogenesis of psoriasis.

Given that MKP-1 is an important negative regulator of MAPKs, we next detected which MAPK signaling in MKP-1^−/−^ macrophages contributed to the dysregulated cytokine production. BMDMs from WT and MKP-1^−/−^ mice were stimulated with R848 at different time points for Western blotting assay. Considering that the activities of ERK and p38 MAPK are increased in lesional psoriatic skin, while JNK activity has been found unaltered in most studies (9, 10), we measured ERK and p38, as well as IκBα activation in R848-stimulated macrophages. We found that p38 activity was enhanced in MKP-1^−/−^ BMDMs, while the IκBα and ERK activation was comparable between WT and MKP-1^−/−^ BMDMs (Figure 4D). To determine whether the increased activity of p38 in MKP-1^−/−^ BMDMs could contribute to the enhanced *Il1b, Cxcl2*, and *S100a8* production, we added p38-specific inhibitor SB203580 to the culture systems. The blockade of p38 largely restored the increased *Il1b, Cxcl2*, and *S100a8* production from MKP-1^−/−^ BMDMs (Figure 4E), suggesting that macrophages may provide signals that promote inflammation in the absence of MKP-1 signaling.

### MKP-1 Deficiency in Non-Hematopoietic Cells Causes Exacerbated Skin Inflammation

**Figure 5:**
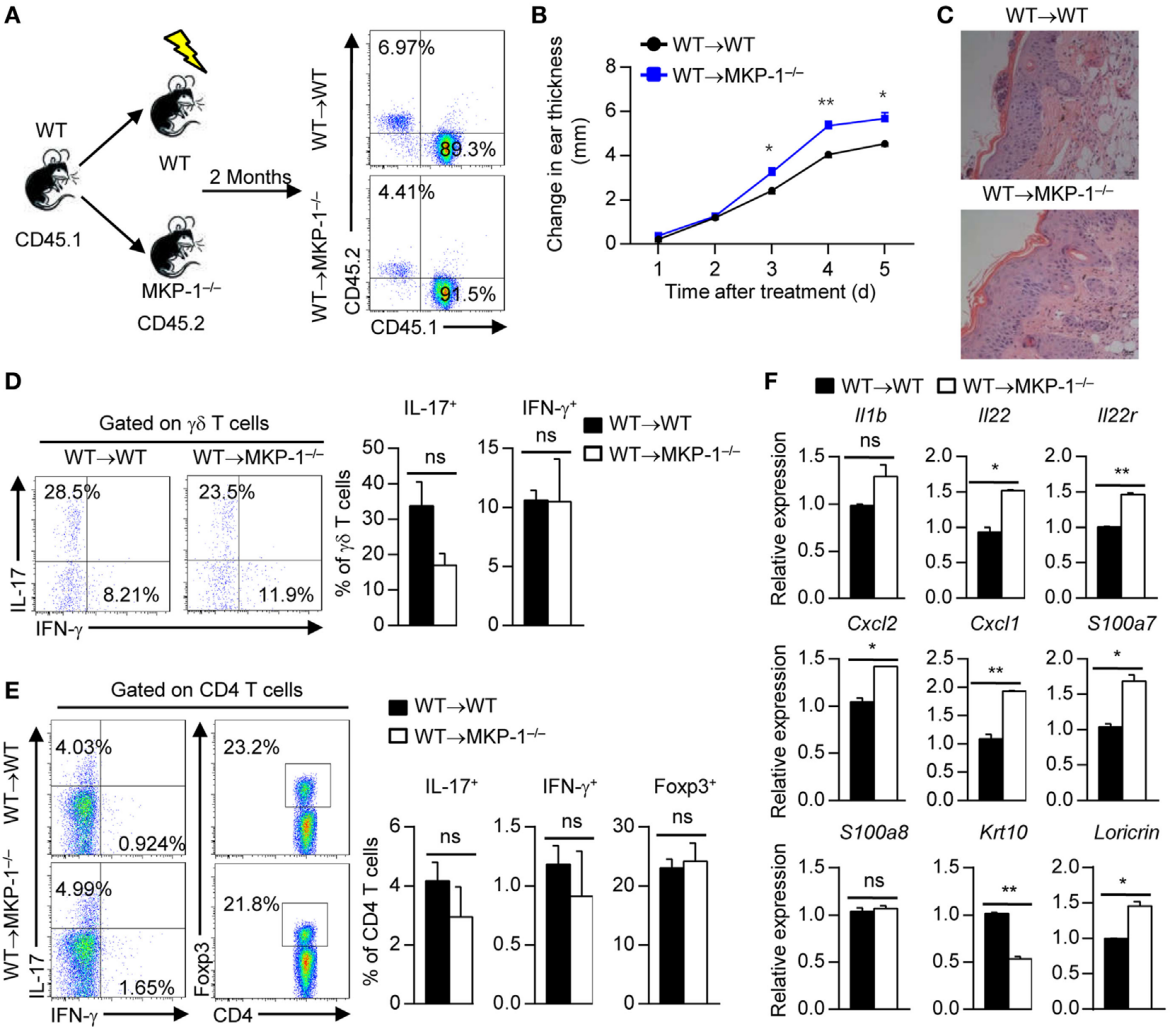
MAPK phosphatase-1 (MKP-1) deficiency in non-hematopoietic cells causes exacerbated skin inflammation. **(A)** Bone marrow (BM) cells of wild-type (WT) mice were, respectively, transplanted into X-ray-irradiated WT and MKP-1^−/−^ mice to generate the WT → WT and WT → MKP-1^−/−^ chimeras. Two months after the generation of BM chimeras, peripheral blood cells were analyzed by flow cytometry. *n* = 5 mice per group. **(B–E)** The chimeras were treated with imiquimod (IMQ) for five consecutive days. Changes in ear thickness **(B)**, representative images of hematoxylin and eosin staining of skin section (scale bars: 50 µm) **(C)**, the percentages of IL-17^+^ γδ T cells and IFN-γ^+^ γδ T cells in the draining lymph nodes (DLNs) **(D)**, and the percentages of IL-17^+^CD4^+^ T cells, IFN-γ^+^CD4^+^ T cells, and Foxp3^+^CD4^+^ T cells in the DLNs **(E)** were analyzed. **(F)** CD45^−^ cells were sorted from the epidermis of IMQ-treated WT and MKP-1^−/−^ mice to examine the cytokine expression. *n* = 3 mice per group. Data are presented as mean ± SEM. Data are representative of two independent experiments.

Having shown that MKP-1 activity in hematopoietic cells dampens the development of IMQ-induced psoriasiform inflammation, to determine whether MKP-1 in non-hematopoietic cells is also critical for psoriasis development, we transplanted WT BM cells into X-ray-irradiated WT and MKP-1^−/−^ mice to generate WT → WT and WT → MKP-1^−/−^ chimeras (Figure 5A). After 2 months, we used IMQ to induce the psoriasiform skin inflammation. We found that IMQ-treated WT → MKP-1^−/−^ chimeras had higher ear thickness than WT → WT chimeras (Figure 5B). Histological analysis also showed that WT → MKP-1^−/−^ chimeras had significantly higher epidermal hyperplasia and inflammation than WT → WT chimeras (Figure 5C). Again, the percentages of IL-17- and IFN-γ-producing γδ^+^ and CD4^+^ T cells were comparable between WT → WT chimeras and WT → MKP-1^−/−^ chimeras (Figures 5D,E).

Given that KCs constitutes 90% of the non-hematopoietic compartment cells in the epidermis, to explore the role of MKP-1 in KCs in the pathogenesis of psoriasis, we sorted CD45^−^ cells (constituting of most of KCs) from IMQ-treated WT and MKP-1^−/−^ mouse epidermis and measured the expressions of cytokines and chemokines. We found that chemokines such as *Cxcl1* and *Cxcl2* were significantly increased in MKP-1^−/−^ KCs compared with WT KCs, while certain pro-inflammatory cytokines such as *Il1b* were comparable between WT and MKP-1^−/−^ KCs (Figure 5F). Moreover, we found an increased expression of certain IL-22 receptor signaling molecules, including *Il22, Il22r*, and *S100a7* in MKP-1^−/−^ KCs compared with WT KCs (Figure 5F). IL-22 has also been shown to inhibit differentiation and maturation of KCs through downregulation of keratin 10 (KRT10) (29). In this study, we found that MKP-1^−/−^ KCs had decreased KRT10 expression compared with WT KCs upon IMQ treatment. Taken together, MKP-1 may contribute to ameliorate psoriasis development in both hematopoietic and non-hematopoietic compartments through regulating different gene expressions.

### Inhibition of p38 Activity Alleviates the Disease Severity in MKP-1^−/−^ Mice

**Figure 6:**
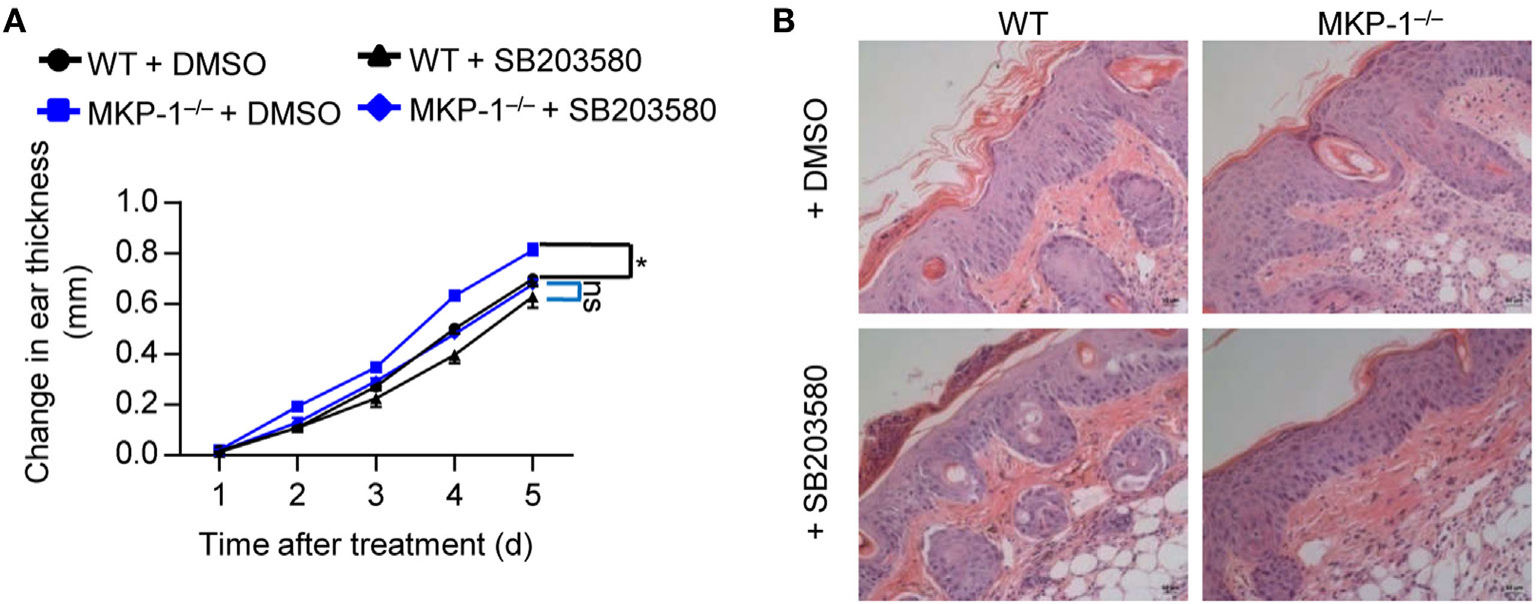
Inhibition of p38 activity alleviates the disease severity of MKP-1^−/−^ mice. Wild-type (WT) and MKP-1^−/−^ mice were topically treated with imiquimod cream for five consecutive days and received p38 inhibitor SB203580 or DMSO (the solvent of Sb203580) *via* intraperitoneally injection daily. *n* = 4–5 mice per group. **(A)** Changes in ear thickness. **(B)** Representative images of hematoxylin and eosin staining in skin section (scale bars: 50 µm). Data are representative of two independent experiments.

To further assess the functional importance of MKP-1-dependent p38 activation *in vivo*, we treated WT and MKP-1^−/−^ mice with IMQ to induce psoriasiform inflammation, and intraperitoneally injected SB203580 or DMSO for five consecutive days. SB203580 injection alleviated the severe ear swelling of both WT and MKP-1^−/−^ mice (Figure 6A), as well as the skin inflammation (Figure 6B). However, the SB203580 treatment largely abolished the difference of disease severity between WT and MKP-1^−/−^ mice (Figures 6A,B). Altogether, these results suggest that MKP-1^−/−^ mice fail to dampen p38 signaling pathways, which ultimately results in elevated cytokine and chemokine levels, and inflammatory responses during psoriasis development.

## Discussion

Persistent activation of MAPK pathway has been suggested to mediate a series of inflammatory diseases, including psoriasis. However, the cellular and molecular mechanisms of the negative regulator of MAPK pathway in psoriasis pathogenesis are still largely unknown. We show here that MKP-1, as a key negative regulator of MAPK pathway, suppresses the development of IMQ-induced psoriasiform skin disease through dampening p38 activation in macrophages. MKP-1^−/−^ mice are hypersusceptible to IMQ-induced psoriasiform skin disease, which was associated with an increased expression of *Il1b, Cxcl2*, and *S100a8*. Chimeric experiments showed that MKP-1 acted on both hematopoietic and non-hematopoietic cells to regulate psoriasis pathogenesis. Inhibition of p38 could largely restore the enhanced skin inflammation in MKP-1^−/−^ mice. Moreover, we found an increased IL-22 receptor signaling in MKP-1^−/−^ KCs upon IMQ treatment. Our results identify a crucial role of MKP-1 in the pathogenesis of chronic inflammatory disorders.

MAPK phosphatase-1 is initially identified as an *in vitro* ERK-specific phosphatase, but later studies showed that it can also dephosphorylate other members of the MAPK family, such as JNK and p38, depending on different cell types (30). In mouse macrophages, the kinetics of ERK1/2 dephosphorylation correlate only with the induction of MKP-1 (31), and the LPS-treated MKP-1^−/−^ macrophages had sustained activation of p38 and JNK (8). However, MKP-1^−/−^ DCs exhibited stronger and more sustained activation of p38, and to a lesser extent, JNK. In this study, when stimulated macrophages with R848, instead of LPS, MKP-1 attenuates the activities of p38, while does not affect ERK activity. Considering that JNK activity has been found unaltered in most psoriasis studies (9, 10), we did not focus on the regulation of JNK by MKP-1 in the current study. Different from previous findings that MKP-1 is essential for the expression TNF-α and IL-10 in LPS-treated macrophages, MKP-1 is found to be not required for the expression of those cytokines upon R848 stimulation, but required for the expression of *Il1b, Cxcl2*, and *S100a8*. Accordingly, inhibition of p38 activity largely restored the increased *Il1b, Cxcl2*, and *S100a8* expression from MKP-1^−/−^ macrophages. These findings indicate that MKP-1 regulating different cytokine expression also depends on different TLR signaling.

Psoriasis is a multiple cell population-involved skin disease (3). Although a comparable cell infiltration and cytokine production of T cells and conventional DCs from both WT and MKP-1^−/−^ mice, this cannot rule out the roles of MKP-1 in DCs and T cells in psoriasis development. Moreover, although macrophages and DCs have been shown to drive psoriatic development, their largely overlapping phenotype makes it difficult to study their respective role (32–35). Indeed, we found that the monocyte-derived inflammatory DCs were also included in this infiltrated macrophage population in the skin, suggesting an important role of MKP-1 in both macrophages and monocyte-derived inflammatory DCs during psoriasis development. Whether the increased macrophages and monocyte-derived inflammatory DCs in MKP-1^−/−^ mice directly contribute to the severe disease is still needed to be elucidated in the future.

Keratinocytes are one of the most important cell types which are involved in psoriasis development (4). However, whether KCs play a role in initiating inflammation or are simply responders to the local cytokines is still controversial. In one hand, KCs are the first line of defense against extracellular stimuli or insults and can secrete large amounts of cytokines and chemokines, which could attract and activate other immune cells to initiate the immune responses in the skin. On the other hand, activation of skin-resident T cells could produce IFN-γ, IL-17, or TNF-α, and these cytokines can act on KCs to promote pro-inflammatory cytokine and chemokine secretion, which further amplify the viciously inflammatory cycle (4). In the current study, although we did not observe the different expressions of IFN-γ, IL-17, or TNF-α in both WT and MKP-1^−/−^ skin tissues during psoriasis development, these cytokines could induce KCs to secrete certain pro-inflammatory cytokines and chemokines, which then amplify specific effector responses. IL-17 has been shown to be a strong inducer for synthesis of AMPs in KCs (36), and IL-22 has diverse effects on KCs, such as induction of proliferation, and secretion of AMPs and chemokines, as well as delays KC differentiation (29, 37). In this study, we found an increased *Il22, Il22r, S100a7, Cxcl1*, and *Cxcl2*, but decreased differentiation molecule *Krt10* in MKP-1^−/−^ KCs upon IMQ treatment. All these changes can cause a switch into an epidermal regenerative growth pathway and faster growth of KCs, which can lead to psoriasis pathogenesis, although more studies still need to be explored.

In conclusion, our data suggest a critical role for MKP-1 in the regulation of skin inflammation. Given that MKP-1 functions as cell type-dependent manner in immunoregulation, selectively targeting MKP-1 might be a useful strategy to treat the inflammatory disorders.

## Ethics Statement

This study was carried out in accordance with the recommendations of the Care and Use of Laboratory Animals with the approval (SYXK-2003-0050) of the Scientific Investigation Board of Shanghai Jiao Tong University School of Medicine, the Institutional Animal Care and Use Committee of Shanghai Jiao Tong University School of Medicine. The protocol was approved by the Institutional Animal Care and Use Committee of Shanghai Jiao Tong University School of Medicine.

## Author Contributions

WZ, SX, and HL designed and performed the *in vivo* and cellular experiments. JH and BZ contributed to gene expression analysis and molecular experiments. WZ and TZ contributed to manuscript writing and data analysis. RH contributed to animal colony management and disease model experiment. XL provided reagents. GH designed experiments, analyzed the data, wrote the manuscript, and provided overall direction.

## Conflict of Interest Statement

The authors declare that the research was conducted in the absence of any commercial or financial relationships that could be construed as a potential conflict of interest.
