# Supplementary material for: MAPK Phosphatase-1 Deficiency Exacerbates the Severity of Imiquimod-Induced Psoriasiform Skin Disease
Source: Front Immunol. 2018 Mar 21;9:569. doi: 10.3389/fimmu.2018.00569 (PMC5873221; doi:10.3389/fimmu.2018.00569)
Supplement: Supplementary file 1 [file Presentation_1.PDF]

Supplementary figure 1. The regulation of MKP-1 expression in different conditions

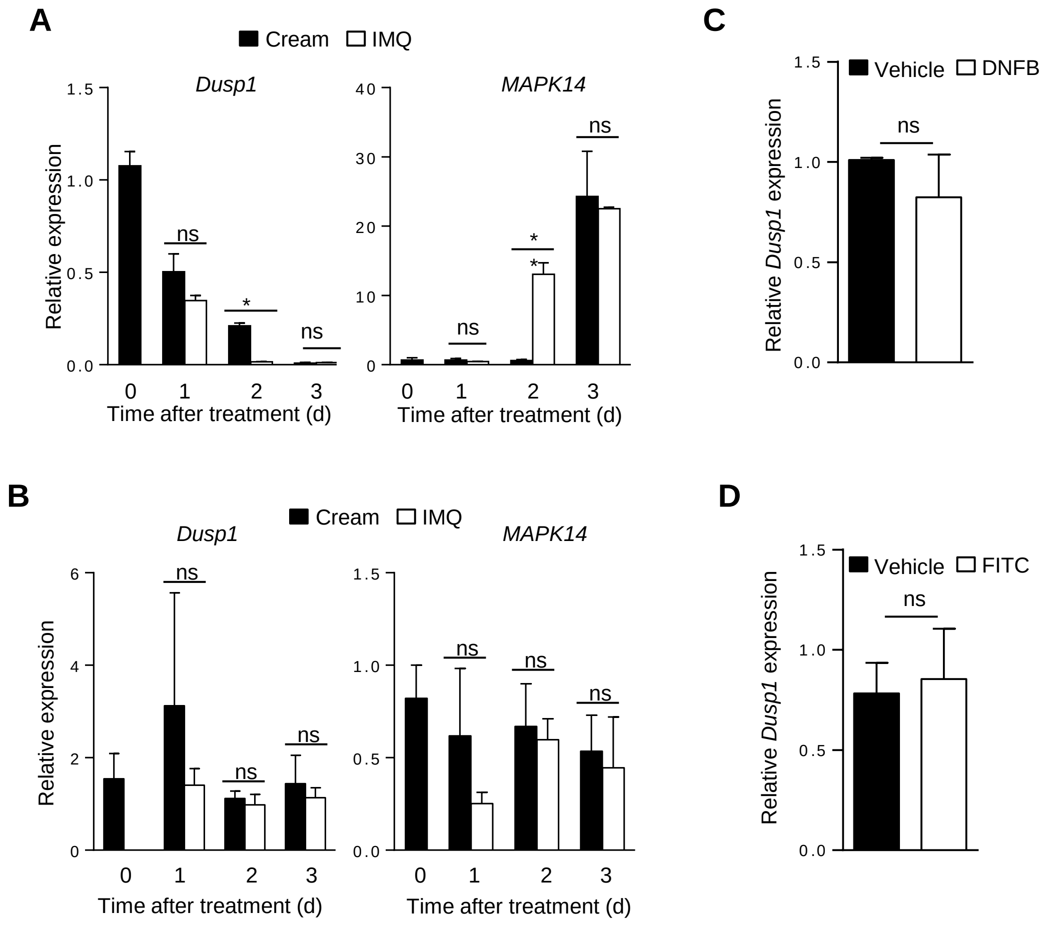

SUPPLEMENTARY FIGURE 1. The regulation of MKP-1 expression in different conditions. (A-B) WT mice were topically treated with IMQ-containing cream or control cream for 0, 1, 2, 3 days and the expression of MKP-1 (*Dusp1*) and p38α (*MAPK14*) was analyzed by real time PCR in inflamed skin tissue (**A**) and DLN tissue (**B**) (n=4 mice/group). **(C and D)** WT mice were topically sensitized with 0.5% DNFB **(C)** or 0.5% FITC **(D)** on shaved skin on days 0 and 1. On day 5, mice were challenged by applying 20 µl 0.5% DNFB or 0.5% FITC . After 24 h, ear tissues were used for the expression of MKP1 mRNA (*Dusp1*) by real-time PCR. (n=5 mice/group). Data are presented as mean ± SEM. Data are representative of two independent experiments. two-sided Student's *t*-tests were performed. * p<0.05,ns, not significant.

Supplementary figure 2. The infiltrated macrophages contain parts of inflammatory monocyte-derived DCs during IMQ treatment in the skin

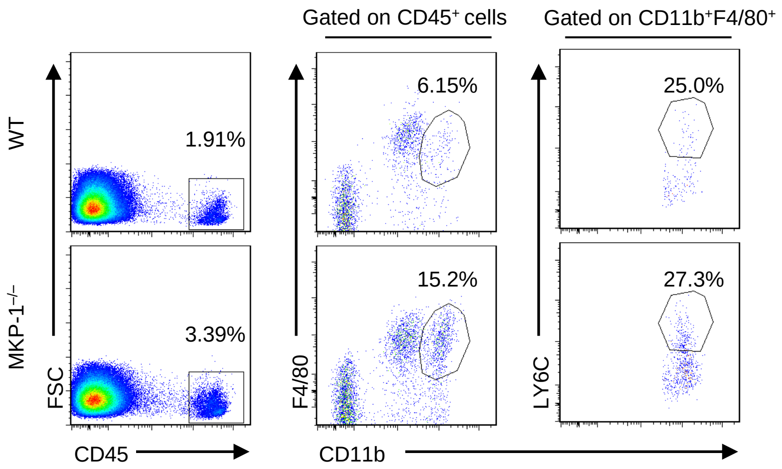

SUPPLEMENTARY FIGURE 2. Cell infiltration analysis in IMQ-treated wild-type and MKP-1$^{-/-}$ mice. Infiltration of indicated cell populations in the epidermis was analyzed by flow cytometry (n=5 mice/group). Data are representative of two independent experiments.
